# Comparing Evolutionary Strategies on a Biobjective Cultural Algorithm

**DOI:** 10.1155/2014/745921

**Published:** 2014-08-31

**Authors:** Carolina Lagos, Broderick Crawford, Enrique Cabrera, Ricardo Soto, José-Miguel Rubio, Fernando Paredes

**Affiliations:** ^1^Escuela de Ingeniería Informática, Pontificia Universidad Católica de Valparaíso, 2362807 Valparaíso, Chile; ^2^Universidad Finis Terrae, 7500000 Santiago, Chile; ^3^CIMFAV Facultad de Ingeniería, Universidad de Valparaíso, 2362735 Valparaíso, Chile; ^4^Universidad Autónoma de Chile, 7500138 Santiago, Chile; ^5^Departamento de Computación e Informática, Universidad de Playa Ancha, 33449 Valparaíso, Chile; ^6^Escuela de Ingeniería Industrial, Universidad Diego Portales, 8370109 Santiago, Chile

## Abstract

Evolutionary algorithms have been widely used to solve large and complex optimisation problems. Cultural algorithms (CAs) are evolutionary algorithms that have been used to solve both single and, to a less extent, multiobjective optimisation problems. In order to solve these optimisation problems, CAs make use of different strategies such as normative knowledge, historical knowledge, circumstantial knowledge, and among others. In this paper we present a comparison among CAs that make use of different evolutionary strategies; the first one implements a historical knowledge, the second one considers a circumstantial knowledge, and the third one implements a normative knowledge. These CAs are applied on a biobjective uncapacitated facility location problem (BOUFLP), the biobjective version of the well-known uncapacitated facility location problem. To the best of our knowledge, only few articles have applied evolutionary multiobjective algorithms on the BOUFLP and none of those has focused on the impact of the evolutionary strategy on the algorithm performance. Our biobjective cultural algorithm, called BOCA, obtains important improvements when compared to other well-known evolutionary biobjective optimisation algorithms such as PAES and NSGA-II. The conflicting objective functions considered in this study are cost minimisation and coverage maximisation. Solutions obtained by each algorithm are compared using a hypervolume S metric.

## 1. Introduction

Evolutionary algorithms (EAs) are an effective alternative to (approximately) solve several large and complex optimisation problems, as they are able to find good solutions for a wide range of problems in acceptable computational time. Although less studied, EAs for multiobjective optimisation (MO) problems, called evolutionary multiobjective optimisation (EMO), have demonstrated to be very effective. In fact, during the last two decades, several authors have focused their efforts on the development of several EMO algorithms to solve a wide range of MO problems. For instance, Maravall and de Lope [1] use a genetic algorithm (GA) to solve the multiobjective dynamic optimisation for automatic parking system. In [2] the authors propose an improvement to the well-known NSGA algorithm (called NSGA-II) based on an elitist approach. In [3] the author presents an EMO algorithm applied on a specific variation of the well-studied capacitated vehicle routing problem (CVRP), where the author includes in the EMO algorithm an explicit collective memory method, namely, the extended virtual loser (EVL). Other well-known EMO algorithms developed during the last two decades are PAES [4] and MO particle swarm optimisation [5]. More recently, hybrid techniques have been also applied to a large number of optimisation problems (see [6]). A comprehensive literature review related to EMO algorithms can be found in [7].

EMO algorithms have some problems that must be taken into account, though. For instance, they tend to fall into premature convergence with low evolution efficiency [8]. This is because of implicit information embodied in the evolution process and domain knowledge corresponding to optimisation problems which are not fully included in the solution approach [9]. To overcome these problems, one can make use of implicit evolution information. Reynolds [10] proposes an EA, called cultural algorithm (CA), which is inspired from human culture evolution process and that makes use of the implicit evolution information generated at each iteration. The CAs have a dual evolution structure which consists of two spaces: population and belief space. On the one hand, the population space works as in any other EA, that is, using evolutionary operators such as mutation and crossover. On the other hand, in the belief space, implicit knowledge is extracted from selected individuals in the population and stored in a different way. Then, they are used to guide the whole evolution process in the population space such that they can induce the population to escape from local optimal solutions. It has been proved that CAs can effectively improve the evolution performance [9]. Although less studied, CAs have been also used to solve MO problems. Coello et al. [11] and Coello et al. [12], two remarkable surveys on CAs, only mention Coello and Landa [13] work as example of a CA application solving MO problems. More recently, Zhang et al. [14] present a CA which is enhanced by using a particle swarm optimisation algorithm. This enhanced CA is applied to fuel distribution MO problem. Srinivasan and Ramakrishnan [15] present a MO cultural algorithm that is applied on data mining domain. In [16] the authors applied a CA to a biobjective portfolio selection problem using normative knowledge in the belief space. In [17] authors present a formal framework to implement MO cultural algorithms. To the best of our knowledge, [18] is the only article that uses CAs to solve the biobjective uncapacitated facility location problem (BOUFLP). Furthermore, we did not find any article which compares the performance of CAs using different evolutionary strategies at the belief space level. Thus, in this paper we present an extension of the biobjective cultural algorithm (BOCA) developed in [18]. We use two different strategies at the belief space level and compare the performance of our new algorithms with the performance of the previous one. We also compare its results with other well-known EMO algorithms such as PAES and NSGA-II. Obtained solutions were compared using the hypervolume S metric proposed in [19].

The remaining of this paper is organised as follows.  Section 2 shows an overview on MO focused on EMO algorithms.  Section 2.1 presents briefly the BOUFLP and some of its distinctive features. In Section 3, we describe our implementation for the BOCA algorithm. We describe the main differences between our implementation and the one in [18].  Section 3.2 presents the BOCA algorithm applied to a set of well-known instances from the literature. Finally, Section 4 presents the conclusions of this work.

## 2. Overview

In this section we show an overview of topics related to this paper. In Section 2.1, MO concepts are presented, emphasizing EMO algorithms and its state of art. More specifically, we focus on the development of EMO algorithms for MO combinatorial optimisation (MOCO) problems. In Section 2.2 we present the BOUFLP formulation based on a cost-coverage approach. Finally, in Section 2.3 we present an overview of CAs and its multiobjective extension. Details of our CA implementation are also presented at the end of this section.

### 2.1. (Evolutionary) Multiobjective Optimisation

**Figure 1:**
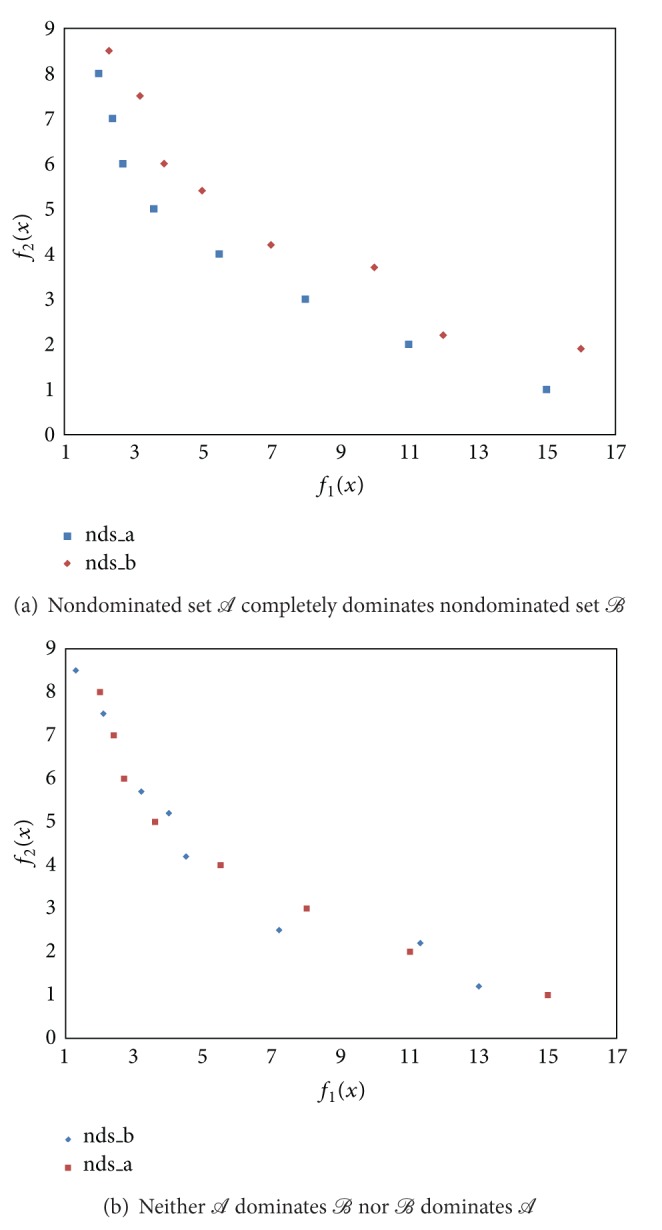
**S**  metric represented as the area that is dominated by a set of (approximately) nondominated points.

In this section we briefly introduce the main principles of MO problems and, particularly, MOCO problems. For a comprehensive review of this topic see [20, 21]. In this paper we will make use of the following notation for the comparison of vectors (solutions). Let *y* and *y*′ ∈ *R* ^*p*^. We say that *y*≦*y*′ if *y*_*k*_≦*y*_*k*_′  ∀*k* = 1,…, *p*. Similarly, we will say that *y* ≤ *y*′ if *y*≦*y*′ but *y* ≠ *y*′. Finally, we say that *y* < *y*′ if *y*_*k*_ < *y*_*k*_′  ∀*k* = 1,…, *p*. A solution $\hat{x}\in R^{n}$, with *n* being equal to the number of decision variables, is called an efficient solution and its image $f(\hat{x})$, with *f* : *R* ^*n*^ → *R* ^*p*^, a nondominated point of the MO problem if there is no *x* ∈ *R* ^*n*^, with $x\neq \hat{x}$, such that $f(x)\leq f(\hat{x})$. In [22] the author describes several excellence relations. These relations establish strict partial orders in the set of all nondominated points related to different aspects of their quality. Previously, in [23, 24] the authors consider several outperformance relations to address the closeness of the set of nondominated points found by an algorithm to the actual set of nondominated points, called Pareto Frontier (PF). In [25] a comprehensive explanation of the desirable features of an approximation to the PF is presented. In this paper, we choose the **S** metric which is properly explained in [24]. The **S** metric calculates the hypervolume of a multidimensional region [19] and allows the integration of aspects that are individually measured by other metrics. An advantage of the **S** metric is that each algorithm can be assessed independently of the other algorithms involved in the study. However, the **S** values of two sets *A* and *B*, namely, **S**_*A*_ and **S**_*B*_, respectively, cannot be used to derive whether either set entirely dominates the other.  Figure 1(a) shows a situation where **S**_*A*_ > **S**_*B*_ and set *A* completely dominates set *B*.  Figure 1(b) shows a situation where **S**_*B*_ > **S**_*A*_ but neither *A* dominates *B* nor *B* dominates *A*.

In this paper we use EAs to solve the BOUFLP. An EA is a stochastic search procedure inspired by the evolution process in nature. In this process, individuals evolve and the fitter ones have a better chance of reproduction and survival. The reproduction mechanisms favour the characteristics of the stronger parents and hopefully produce better children guaranteeing the presence of those characteristics in future generations [26]. EAs have been successfully applied to a large number of both single- and multiobjective optimisation problems. Comprehensive reviews of EMO algorithms are presented in [11, 24] and more recently in [12]. A more general review of hybrid heuristics solving MOCO problems, where EMO algorithms are also included, is presented in [27].

### 2.2. Biobjective Uncapacitated Facility Location Problem

Facility location problem (FLP) is one of the most important problems for companies with the aim of distributing products to their customers. The problem consists of selecting sites to install plants, warehouses, and distribution centres, allocating customers to serving facilities and interconnecting facilities by flow assignment decisions. Comprehensive reviews and analysis of the FLP are presented in [28–30].

**Figure 2:**
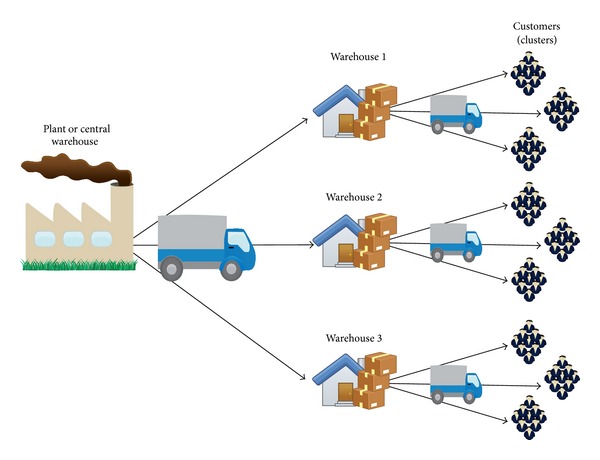
A two-level supply chain network configuration.

In this paper we consider a two-level supply chain, where a single plant serves a set of warehouses, which serve a set of end customers or retailers.  Figure 2 shows this configuration.

Two main (conflicting) objectives can be identified in the FLP:

- minimise the total cost associated with the facility installation and customer allocation and
- maximise the customers rate coverage.

Several works on single-objective optimisation have been carried out considering these two objectives separately. On the one hand, uncapacitated FLP (UFLP) is one of most studied FLPs in the literature. In the UFLP the main goal is to minimise the location-allocation cost of the network. On the other hand, *p* median FLPs are one of the most common FLPs among those that are focused on coverage maximisation. Most important FLP models are well described and formalised in [29]. MO FLPs have been also well studied in the literature during the last two decades. A survey on this topic can be found in [31].

As we mentioned before, in this paper we solve the BOUFLP. BOUFLP has been modelled with minisum and maxisum objectives (cost and coverage). The following model formulation is based on [32]. Let *I* = {1,…, *m*} be the set of potential facilities and *J* = {1,…, *n*} the set of customers. Let FC_*i*_ be the fixed cost of opening facility *i* and *d*_*j*_ the demand of customer *j*. Let *c*_*ij*_ be the cost of assigning the customer *j* to facility *i* and *h*_*ij*_ the distance between facility *i* and customer *j*. Let *D*_MAX_ be the maximal covering distance; that is, customers within this distance to an open facility are considered well served. Let *Q* ^*j*^ = {*i* ∈ *I* : *h*_*ij*_ ≤ *D*_MAX_} be the set of facilities that could serve customer *j* within the maximal covering distance *D*_MAX_. Let *x*_*i*_ be 1 if facility *i* is open and 0, otherwise. Let *y*_*ij*_ be 1 if the whole demand of customer *j* is served by facility *i* and 0, otherwise. Consider

(1)   minimise f(x,y)

(2)   s.t. ∑i∈Iyij ∀j∈J

(3) $$\begin{array}{c} \;y_{ij}\leq x_{i}\;\forall j\in J,\;\;i\in I \end{array}$$

(4) $$\begin{array}{c} \;y_{ij}\in \left\{0,1\right\}\;\forall j\in J,i\in I \end{array}$$

(5) $$\begin{array}{c} \;x_{i}\in \left\{0,1\right\}\;\forall i\in I \end{array}$$

with *f* : *R* ^*n*^ → *R* ^*p*^ and *p* = 2. Objective functions *f*_1_ and *f*_2_ are as follows:

(6) $$\begin{array}{c} f_{1}\left(x,y\right)=\underset{i\in I}{\sum }{\text{FC}}_{i}x_{i}+\underset{i\in I}{\sum }\underset{j\in J}{\sum }c_{ij}y_{ij}, \end{array}$$

(7) $$\begin{array}{c} f_{2}\left(x,y\right)=-\underset{j\in J}{\sum }d_{j}\underset{i\in Q^{j}}{\sum }y_{ij}. \end{array}$$

Equation (6) represents total operating cost; the first term corresponds to location cost, that is, the sum of the fixed costs of all the open facilities, and the second term represents the allocation cost, that is, the cost of attending customer demand by an open facility. Equation (7) measures coverage as the sum of the demand of customers attended by open facilities within the maximal covering distance. Equations (2) and (3) ensure that each customer is attended by only one facility. Equation (3) also forces customer to be assigned to an open facility. Finally, equations (4) and (5) set decision variables as binary.

### 2.3. Biobjective Cultural Algorithm

**Figure 3:**
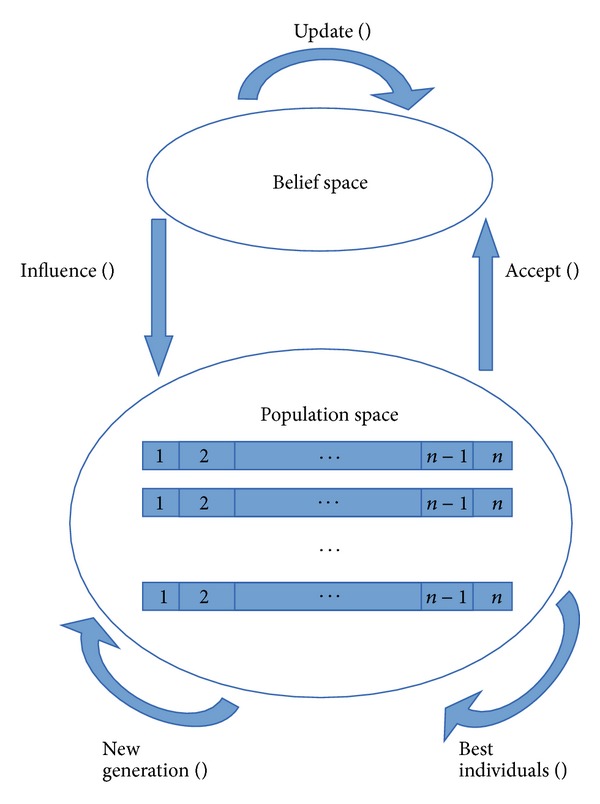
CA general diagram.

The experience and beliefs accepted by a community in a social system are the main motivations for the creation of the CAs. Originally proposed by Reynolds [10], CAs model the evolution of cultural systems based on the principles of human social evolution. In this case, evolution is seen as an optimisation process [10]. The CAs guide the evolution of the population based on the knowledge. Knowledge acquired during previous iterations is provided to future generations, allowing accelerating the convergence of the algorithm to good solutions [33]. Domain knowledge is modelled separately from the population, because there is certain independence between them which allows us to work and model them separately, in order to enhance the overall algorithm performance.  Figure 3 shows this interaction.

CAs are mainly characterised by presenting two inheritance systems: one at population level, called* population space,* and the other at knowledge level, called* belief space*. This key feature is designed to increase the learning rates and convergence of the algorithm and thus to do a more responsive system for a number of problems [34]. Moreover, it allows us to identify two significant levels of knowledge: a microevolutionary level (represented by the population space) and macroevolutionary level (represented by the space of beliefs) [35].

CAs have the following components: population space (set of individuals who have independent features) [35]; belief space (stored individuals acquired in previous generations) [34]; computer protocol, connecting the two spaces and defining the rules on the type of information to be exchanged between them by using the acceptance and influence functions; and finally knowledge sources which are described in terms of their ability to coordinate the distribution of individuals depending on the nature of a problem instance [35]. These knowledge sources can be of the following types: circumstantial, normative, domain, topographic, and historical.

The most distinctive feature of CAs is the use of the belief space which through an influence function affects future generations. For this reason, in this paper we focus on the effect on the algorithm performance of changes in such an influence function. To do this, we have considered results obtained previously in [18], where the authors used an influence function based on historical knowledge, and we compare those results with our BOCA implementation which considers two influence functions: the first one based on circumstantial knowledge and the second one based on normative knowledge.  Algorithm 1 shows the general procedure of our BOCA algorithm.

To initialise the population, we use a semirandom function. In its first phase, this function defines in a stochastic way the set of facilities that will be opened (selected facilities). Then, we allocate each customer to a selected facility minimising the cost function *f*_1_ while avoiding minimising the coverage function *f*_2_. This strategy provides better results than using completely random initial populations, and its computational time additional cost is marginal.

To obtain the next generation, two parents are used in a recombination process. To avoid local optimal values, we do not overuse the culture. Thus, a parent is selected from the population to obtain diversity and the other parent is selected from the belief space to influence the next generation. The belief space keeps a list of all the individuals which meet some criteria. These criteria depend on what knowledge the algorithm implements. In this paper the circumstantial knowledge selects the best individuals found so far for each objective function. Thus, one individual will give us information on the best value found for *f*_1_ and the other will do the same for *f*_2_. The historical knowledge stores a list of individuals with the best fitness value found so far. The fitness value is calculated as the hypervolume **S** that is covered by an individual. Finally, normative knowledge considers a list of individuals which are pairwise nondominated with respect to the other individuals of their generation.

Let |*J*| be the number of available facilities and let |*I*| be the number of customers of our BOUFLP. In this paper decisions variables *x* and *y* are represented by a binary |*J*|-length vector and |*J* | ×|*I*| matrix, respectively. Comparing two different solutions (individuals) needs an evaluation criterion. In this paper we use the same criterion explained in [18].

## 3. Computational Experiments

In this section we present the set of instances that are used in this study as well as results obtained by our BOCA implementation.

### 3.1. Instances Presentation

The instances that were used in this paper correspond to random instances using a problem generator that follows the methodology from UflLib [36]. Previous works in the literature have also used this problem generator to create their test instances [18, 26].

The BOCA algorithm has several parameters that need to be set. As in [18], the number of generations considered in this paper is equal to 100. Population size *L* is set equal to 100, mutation probability in the population space *P*_ps_ is equal to 0.2, and probability of mutation in the belief space *P*_bs_ is 0.04. Both *L* and *P*_ps_ values are different from the values used in [18]. These values are chosen as they all together yield to the best performance of the algorithm given some test instances. Thus, although resulting values are different from that used in [18], the method we use to set them is the same as that used in that work. This is important in order to fairly compare the different BOCA algorithms.

### 3.2. Results and Discussion

In this section we compare the results obtained by the previous BOCA algorithm (see [18] for further details) and our approach. Moreover, a comparison between results obtained by well-known EMO algorithms such as NSGA-II and PAES and our BOCA algorithm is also presented in this section.

Tables 1 and 2 show the results obtained by the BOCA implementations using historical [18], circumstantial, and normative knowledge, respectively. In the same way, Tables 3 and 4 present the results obtained by the well-known NSGA-II and PAES algorithms. For each algorithm *S* value (%), time *t* (in seconds), and the number of efficient solutions $\hat{x}\in \hat{X}$ have been included in these tables. As we mentioned before, we want to produce a set with a large number of efficient solutions $\hat{x}\in \hat{X}$, a *S* value close to 100% (ideal), and a small *t*. For the sake of easy reading, we have split the set of instances into two subsets (instances type *A* and *B*).

We then compare our BOCA implementations with the one presented in [18]. Tables 5 and 6 show a comparison between those algorithms. As we can see, when compared in terms of its *S* value (the bigger, the better), BOCA algorithm using historical knowledge (BOCA^H^) performs consistently better than the ones using circumstantial (BOCA^C^) and normative (BOCA^N^) knowledge. In fact BOCA^H^ obtains a *S* value that is, in average, 5.8% bigger than the one obtained by BOCA^C^ and 6.5% bigger than the *S* value obtained by BOCA^N^. When compared in terms of the CPU time needed to reach the number of iterations (generations), BOCA^H^ is, in average, faster than both BOCA^C^ and BOCA^N^ algorithms. We can note that for *B* instances, times required by BOCA^H^ and BOCA^C^ are, in average, quite similar (only 1.6% of difference). Finally, when we look at the number of efficient solutions found by each algorithm ($\left|\hat{X}\right|$ column), we can see that, again, BOCA^H^ outperforms both BOCA^C^ and BOCA^N^ algorithms. In this case, the average number of efficient solutions found by the BOCA^H^ algorithm is about 20% bigger than the one obtained by the other two approaches.

Results above are consistent with the good performance obtained by the BOCA^H^ approach in [18]. Moreover, results show that performance of the BOCA algorithm depends largely on the selected knowledge and it can make the difference in terms of *S* value, time, and number of efficient solutions found by the algorithm. This is an important finding as it points out the relevance of the choice of a specific type of knowledge.

We now compare BOCA^C^ and BOCA^N^ algorithms to the well-known NSGA-II and PAES algorithms. Tables 7 and 8 show a comparison between our BOCA^C^ algorithm and the NSGA-II and PAES algorithms. As we can see, although BOCA^C^ obtains, in average, a *S* value 6.8% lower than the one obtained by the NSGA-II algorithm, it is more than three times faster. Moreover, when BOCA^C^ is compared to PAES algorithm, the obtained *S* values are, in average, equivalent while BOCA^C^ is around 30% faster than PAES. PAES obtains, in average, more efficient points than BOCA^C^ though (9.32%).

Finally, Tables 9 and 10 show a comparison between BOCA^N^ and NSGA-II and PAES algorithms. BOCA^N^ performs quite similar to PAES algorithm with respect to both *S* value and the number of obtained efficient solutions. However, BOCA^N^ is faster than PAES. Similar situation occurs when BOCA^N^ is compared to NSGA-II algorithm. Although NSGA-II obtains better values for both *S* and $\left|\hat{X}\right|$, BOCA^N^ is much faster than NSGA-II. This situation can be explained by the very fast performance that our BOCA^N^ algorithm obtains for the set of small instances. When we look further at the results, we can note that if we only consider both medium and large size instances, execution times obtained by both algorithms are quite similar to each other. This result confirms what is outlined in [18] in the sense of the good performance that the BOCA algorithm shows. Furthermore, our results confirm this good performance with respect to other well-known EMO algorithms does not depend on which type of knowledge is considered. However, as we mentioned before, the choice of the knowledge used on the BOCA algorithm is an important issue and it has an impact on the algorithm performance.

## 4. Conclusions and Future Work

Evolutionary algorithms are a very good alternative to solve complex combinatorial optimisation problems. In this paper we have implemented a biobjective cultural algorithm to solve the well-known BOUFLP. We have considered two different sources of knowledge, namely, circumstantial and normative, and compare them with a previously implemented historical knowledge. Furthermore, we compare our BOCA approaches with two well-known EAs, namely, NSGA-II and PAES.

Although BOCA approaches using both normative and circumstantial knowledge could not improve the results obtained by the BOCA algorithm with the historical knowledge, results pointed out that performance of the BOCA algorithm depends largely on the selected knowledge and it can make the difference in terms of *S* value, time, and number of efficient solutions found by the algorithm. This is an important finding as it points out the relevance of the choice of a specific type of knowledge. Moreover, our results also confirm the good performance showed by the BOCA algorithm with respect to other well-known EMO algorithms such as NSGA-II and PAES algorithms. The BOCA algorithm is very competitive when compared to those EMO algorithms independently of the type of knowledge implemented.

As a future work we think that more investigation is needed in order to find patterns that allow us to get the right knowledge implemented depending on the problem features. As we mentioned before, the knowledge choice has an impact on the performance of the BOCA algorithm and therefore it must be studied in depth. Also, as future work, hybrid knowledge could be implemented in order to exploit the advantages of each kind of knowledge at the same time. Moreover, our BOCA algorithm can be used to solve other interesting MOPs arising in the logistic field, such as routing or scheduling problems.

## Figures and Tables

**Algorithm 1:**
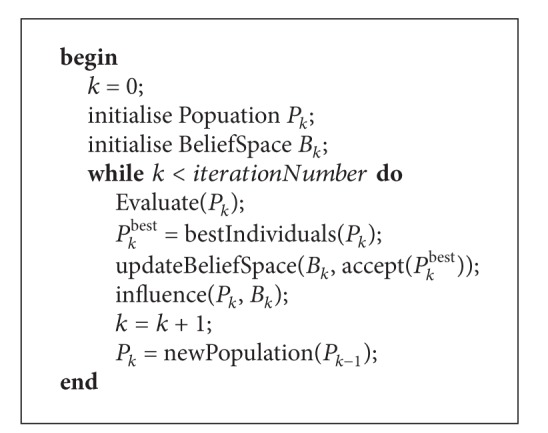
BOCA general algorithmic frame.

**Table 1:** Results obtained by the BOCA implementations for instance of class *A*.

Instance	BOCA (historical)			BOCA (circumstantial)			BOCA (normative)		
	*t*_1_ (sec)	**S**_1_ %	$\left|{\hat{X}}_{1}\right|$	*t*_2_ (sec)	**S**_2_ %	$\left|{\hat{X}}_{2}\right|$	*t*_3_ (sec)	**S**_3_ %	$\left|{\hat{X}}_{3}\right|$
*A*_10–25_ *C*_1_	252	0.7057	13	167	0.7058	13	37	0.6963	11
*A*_10–25_ *C*_2_	197	0.6136	11	176	0.6136	11	27	0.6135	10
*A*_10–25_ *C*_3_	186	0.6993	9	184	0.6992	9	33	0.6992	9
*A*_10–25_ *C*_4_	115	0.5729	5	193	0.5729	5	28	0.5728	3
*A*_10–25_ *C*_5_	152	0.6351	8	168	0.6352	8	32	0.6352	8
*A*_10–25_ *C*_6_	183	0.5982	9	186	0.5961	8	34	0.5944	8
*A*_30–75_ *C*_1_	464	0.8071	18	793	0.7828	16	1908	0.7300	12
*A*_30–75_ *C*_2_	1247	0.8013	47	598	0.7798	23	1201	0.7090	30
*A*_30–75_ *C*_3_	1177	0.7552	50	611	0.7374	21	1305	0.7098	29
*A*_30–75_ *C*_4_	450	0.7471	21	960	0.6307	10	1450	0.6048	15
*A*_30–75_ *C*_5_	940	0.7790	46	610	0.7628	32	1725	0.7078	28
*A*_30–75_ *C*_6_	740	0.7245	37	536	0.6656	14	446	0.7010	28
*A*_50–150_ *C*_1_	1473	0.7878	52	2386	0.6685	27	3481	0.7140	42
*A*_50–150_ *C*_2_	1043	0.7611	37	4266	0.6418	27	5412	0.6200	32
*A*_50–150_ *C*_3_	1165	0.7883	53	1644	0.7171	24	3941	0.7160	27
*A*_50–150_ *C*_4_	735	0.6345	17	1437	0.5849	17	3419	0.6010	20
*A*_50–150_ *C*_5_	1459	0.8039	53	2333	0.6725	21	2745	0.7158	35
*A*_50–150_ *C*_6_	1434	0.7903	43	2399	0.6535	26	2811	0.7040	48

**Table 2:** Results obtained by the BOCA implementations for instance of class *B*.

Instance	BOCA (historical)			BOCA (circumstantial)			BOCA (normative)		
	*t*_1_ (sec)	**S**_1_ %	$\left|{\hat{X}}_{1}\right|$	*t*_2_ (sec)	**S**_2_ %	$\left|{\hat{X}}_{2}\right|$	*t*_3_ (sec)	**S**_3_ %	$\left|{\hat{X}}_{3}\right|$
*B*_10–25_ *C*_1_	135	0.7795	7	196	0.7795	7	36	0.7721	8
*B*_10–25_ *C*_2_	130	0.5856	5	128	0.5813	4	48	0.5807	4
*B*_10–25_ *C*_3_	238	0.7410	14	229	0.7278	12	101	0.7170	13
*B*_10–25_ *C*_4_	260	0.7261	14	222	0.7246	14	182	0.7218	12
*B*_10–25_ *C*_5_	115	0.8117	6	185	0.7250	4	25	0.8020	5
*B*_10–25_ *C*_6_	285	0.6517	18	245	0.6340	15	31	0.5967	12
*B*_30–75_ *C*_1_	683	0.7650	38	736	0.6945	26	1208	0.7119	20
*B*_30–75_ *C*_2_	944	0.7323	59	882	0.7133	30	1014	0.7115	24
*B*_30–75_ *C*_3_	1351	0.6335	72	683	0.6263	28	1076	0.6106	28
*B*_30–75_ *C*_4_	618	0.5214	32	857	0.4947	27	472	0.4824	29
*B*_30–75_ *C*_5_	905	0.7473	45	917	0.6869	37	1820	0.6647	25
*B*_30–75_ *C*_6_	800	0.8775	42	565	0.8502	24	781	0.8310	35
*B*_50–150_ *C*_1_	802	0.8345	23	885	0.8105	18	2371	0.8090	20
*B*_50–150_ *C*_2_	1221	0.7634	53	1405	0.7134	24	2678	0.7050	35
*B*_50–150_ *C*_3_	1410	0.7915	58	1706	0.7169	13	2791	0.6940	45
*B*_50–150_ *C*_4_	567	0.7498	10	604	0.6720	17	3841	0.6420	15
*B*_50–150_ *C*_5_	939	0.6393	37	482	0.5470	27	5712	0.5870	25
*B*_50–150_ *C*_6_	1904	0.8016	67	1745	0.7560	34	4958	0.7150	54

**Table 3:** Results obtained by well-known MOEA algorithms NSGA-II and PAES for instances of class *A*.

Instance	NSGA-II			PAES		
	*t*_4_ (sec)	**S**_4_ %	$\left|{\hat{X}}_{4}\right|$	*t*_5_ (sec)	**S**_5_ %	$\left|{\hat{X}}_{5}\right|$
*A*_10–25_ *C*_1_	1344	0.7057	13	372	0.7057	13
*A*_10–25_ *C*_2_	1511	0.6136	11	394	0.6136	11
*A*_10–25_ *C*_3_	1859	0.6993	9	326	0.6993	9
*A*_10–25_ *C*_4_	3186	0.5729	5	305	0.5729	5
*A*_10–25_ *C*_5_	1748	0.6351	8	378	0.6351	8
*A*_10–25_ *C*_6_	1650	0.5982	9	384	0.5982	9
*A*_30–75_ *C*_1_	1633	0.8071	18	499	0.7795	16
*A*_30–75_ *C*_2_	1345	0.8012	43	622	0.7915	41
*A*_30–75_ *C*_3_	1394	0.7538	43	603	0.7384	29
*A*_30–75_ *C*_4_	1874	0.7508	20	525	0.7342	20
*A*_30–75_ *C*_5_	1413	0.7783	40	595	0.7572	33
*A*_30–75_ *C*_6_	1474	0.6676	36	511	0.5300	12
*A*_50–150_ *C*_1_	2385	0.7913	43	1386	0.7391	25
*A*_50–150_ *C*_2_	2522	0.7597	39	1231	0.6729	23
*A*_50–150_ *C*_3_	2298	0.7665	34	1269	0.6900	26
*A*_50–150_ *C*_4_	2575	0.6344	17	1233	0.6106	15
*A*_50–150_ *C*_5_	2446	0.8072	40	1251	0.7590	23
*A*_50–150_ *C*_6_	2259	0.7862	38	1218	0.6461	27

**Table 4:** Results obtained by well-known MOEA algorithms NSGA-II and PAES for instances of class *B*.

Instance	NSGA-II			PAES		
	*t*_4_ (sec)	**S**_4_ %	$\left|{\hat{X}}_{4}\right|$	*t*_5_ (sec)	**S**_5_ %	$\left|{\hat{X}}_{5}\right|$
*B*_10–25_ *C*_1_	1439	0.7795	7	397	0.7795	7
*B*_10–25_ *C*_2_	1430	0.5856	5	405	0.5856	5
*B*_10–25_ *C*_3_	1288	0.741	13	433	0.7410	13
*B*_10–25_ *C*_4_	1747	0.7261	14	440	0.7261	14
*B*_10–25_ *C*_5_	1766	0.8117	6	363	0.8117	6
*B*_10–25_ *C*_6_	1261	0.6517	17	394	0.6517	17
*B*_30–75_ *C*_1_	1566	0.7609	30	562	0.7134	27
*B*_30–75_ *C*_2_	1525	0.7285	49	563	0.6899	34
*B*_30–75_ *C*_3_	1345	0.6330	51	578	0.5991	53
*B*_30–75_ *C*_4_	2562	0.5333	23	502	0.5211	21
*B*_30–75_ *C*_5_	1433	0.7631	31	564	0.7360	26
*B*_30–75_ *C*_6_	1460	0.8088	47	585	0.7602	32
*B*_50–150_ *C*_1_	2698	0.8447	23	1280	0.6925	10
*B*_50–150_ *C*_2_	2289	0.7515	41	1241	0.4970	13
*B*_50–150_ *C*_3_	2284	0.7988	52	1279	0.6836	26
*B*_50–150_ *C*_4_	2603	0.7500	10	1192	0.7253	14
*B*_50–150_ *C*_5_	2301	0.6387	35	1212	0.5021	16
*B*_50–150_ *C*_6_	3178	0.8029	43	1260	0.7152	32

**Table 5:** Comparison among our BOCA implementations (*A* instances).

Instance	Δ_**S**_2__^**S**_1_^	Δ_*t*_2__^*t*_1_^	$\Delta_{\left|{\hat{X}}_{2}\right|}^{\left|{\hat{X}}_{1}\right|}$	Δ_**S**_3__^**S**_1_^	Δ_*t*_3__^*t*_1_^	$\Delta_{\left|{\hat{X}}_{3}\right|}^{\left|{\hat{X}}_{1}\right|}$
*A*_10−25_ *C*_1_	−0.014	33.73	0.00	1.332	85.32	15.38
*A*_10−25_ *C*_2_	0.000	10.66	0.00	0.016	86.29	9.09
*A*_10−25_ *C*_3_	0.014	1.08	0.00	0.014	82.26	0.00
*A*_10−25_ *C*_4_	0.000	−67.83	0.00	0.017	75.65	40.00
*A*_10−25_ *C*_5_	−0.016	−10.53	0.00	−0.016	78.95	0.00
*A*_10−25_ *C*_6_	0.351	−1.64	11.11	0.635	81.42	11.11
*A*_30−75_ *C*_1_	3.011	−70.91	11.11	9.553	−311.21	33.33
*A*_30−75_ *C*_2_	2.683	52.04	51.06	11.519	3.69	36.17
*A*_30−75_ *C*_3_	2.357	48.09	58.00	6.012	−10.88	42.00
*A*_30−75_ *C*_4_	15.58	−113.33	52.38	19.047	−222.22	28.57
*A*_30−75_ *C*_5_	2.080	35.11	30.43	9.140	−83.51	39.13
*A*_30−75_ *C*_6_	8.130	27.57	62.16	3.244	39.73	24.32
*A*_50−150_ *C*_1_	15.143	−61.98	48.08	9.368	−136.32	19.23
*A*_50−150_ *C*_2_	15.675	−309.01	27.03	18.539	−418.89	13.51
*A*_50−150_ *C*_3_	9.0320	−41.12	54.72	9.172	−238.28	49.06
*A*_50−150_ *C*_4_	7.8170	−95.51	0.00	5.280	−365.17	−17.65
*A*_50−150_ *C*_5_	16.345	−59.90	60.38	10.959	−88.14	33.96
*A*_50−150_ *C*_6_	17.310	−67.29	39.53	10.920	−96.03	−11.63

**Table 6:** Comparison among our BOCA implementations.

Instance	Δ_**S**_2__^**S**_1_^	Δ_*t*_2__^*t*_1_^	$\Delta_{\left|{\hat{X}}_{2}\right|}^{\left|{\hat{X}}_{1}\right|}$	Δ_**S**_3__^**S**_1_^	Δ_*t*_3__^*t*_1_^	$\Delta_{\left|{\hat{X}}_{3}\right|}^{\left|{\hat{X}}_{1}\right|}$
*B*_10–25_ *C*_1_	0.000	−45.19	0.00	0.949	73.33	−14.29
*B*_10–25_ *C*_2_	0.734	1.54	20.00	0.837	63.08	20.00
*B*_10–25_ *C*_3_	1.781	3.78	14.29	3.239	57.56	7.14
*B*_10–25_ *C*_4_	0.207	14.62	0.00	0.592	30.00	14.29
*B*_10–25_ *C*_5_	10.681	−60.87	33.33	1.195	78.26	16.67
*B*_10–25_ *C*_6_	2.716	14.04	16.67	8.439	89.12	33.33
*B*_30–75_ *C*_1_	9.216	−7.76	31.58	6.941	−76.87	47.37
*B*_30–75_ *C*_2_	2.595	6.57	49.15	2.840	−7.42	59.32
*B*_30–75_ *C*_3_	1.137	49.44	61.11	3.615	20.36	61.11
*B*_30–75_ *C*_4_	5.121	−38.67	15.63	7.480	99.92	9.38
*B*_30–75_ *C*_5_	8.082	−1.33	17.78	11.053	−101.10	44.44
*B*_30–75_ *C*_6_	3.111	29.38	42.86	5.299	2.38	16.67
*B*_50–150_ *C*_1_	2.876	−10.35	21.74	3.056	−195.64	13.04
*B*_50–150_ *C*_2_	6.550	−15.07	54.72	7.650	−119.33	33.96
*B*_50–150_ *C*_3_	9.425	−20.99	77.59	12.318	−97.94	22.41
*B*_50–150_ *C*_4_	10.376	−6.53	−70.00	14.377	−577.43	−50.00
*B*_50–150_ *C*_5_	14.438	48.67	27.03	8.181	−508.31	32.43
*B*_50–150_ *C*_6_	5.689	8.35	49.25	10.803	−160.40	19.40

**Table 7:** Comparison among our BOCA with circumstantial knowledge and NSGA-II and PAES.

Instance	Δ_**S**_4__^**S**_2_^	Δ_*t*_4__^*t*_2_^	$\Delta_{\left|{\hat{X}}_{4}\right|}^{\left|{\hat{X}}_{2}\right|}$	Δ_**S**_5__^**S**_2_^	Δ_*t*_5__^*t*_2_^	$\Delta_{\left|{\hat{X}}_{5}\right|}^{\left|{\hat{X}}_{2}\right|}$
*A*_10–25_ *C*_1_	−1.35	−3532.43	−18.18	−1.35	−905.41	−18.18
*A*_10–25_ *C*_2_	−0.02	−5496.30	−10.00	−0.02	−1359.26	−10.00
*A*_10–25_ *C*_3_	−0.01	−5533.33	0.00	−0.01	−887.88	0.00
*A*_10–25_ *C*_4_	−0.02	−11278.57	−66.67	−0.02	−989.29	−66.67
*A*_10–25_ *C*_5_	0.02	−5362.50	0.00	0.02	−1081.25	0.00
*A*_10–25_ *C*_6_	−0.64	−4752.94	−12.50	−0.64	−1029.41	−12.50
*A*_30–75_ *C*_1_	−10.56	14.41	−50.00	−6.78	73.85	−33.33
*A*_30–75_ *C*_2_	−13.00	−11.99	−43.33	−11.64	48.21	−36.67
*A*_30–75_ *C*_3_	−6.20	−6.82	−48.28	−4.03	53.79	0.00
*A*_30–75_ *C*_4_	−24.14	−29.24	−33.33	−21.40	63.79	−33.33
*A*_30–75_ *C*_5_	−9.96	18.09	−42.86	−6.98	65.51	−17.86
*A*_30–75_ *C*_6_	4.76	−230.49	−28.57	24.39	−14.57	57.14
*A*_50–150_ *C*_1_	−10.83	31.49	−2.38	−3.52	60.18	40.48
*A*_50–150_ *C*_2_	−22.53	53.40	−21.88	−8.53	77.25	28.13
*A*_50–150_ *C*_3_	−7.05	41.69	−25.93	3.63	67.80	3.70
*A*_50–150_ *C*_4_	−5.56	24.69	15.00	−1.60	63.94	25.00
*A*_50–150_ *C*_5_	−12.77	10.89	−14.29	−6.04	54.43	34.29
*A*_50–150_ *C*_6_	−11.68	19.64	20.83	8.22	56.67	43.75

**Table 8:** Comparison among our BOCA with circumstantial knowledge and NSGA-II and PAES.

Instance	Δ_**S**_4__^**S**_2_^	Δ_*t*_4__^*t*_2_^	$\Delta_{\left|{\hat{X}}_{4}\right|}^{\left|{\hat{X}}_{2}\right|}$	Δ_**S**_5__^**S**_2_^	Δ_*t*_5__^*t*_2_^	$\Delta_{\left|{\hat{X}}_{5}\right|}^{\left|{\hat{X}}_{2}\right|}$
*B*_10–25_ *C*_1_	−0.96	−3897.22	12.50	−0.96	−1002.78	12.50
*B*_10–25_ *C*_2_	−0.84	−2879.17	−25.00	−0.84	−743.75	−25.00
*B*_10–25_ *C*_3_	−3.35	−1175.25	0.00	−3.35	−328.71	0.00
*B*_10–25_ *C*_4_	−0.60	−859.89	−16.67	−0.60	−141.76	−16.67
*B*_10–25_ *C*_5_	−1.21	−6964.00	−20.00	−1.21	−1352.00	−20.00
*B*_10–25_ *C*_6_	−9.22	−3967.74	−41.67	−9.22	−1170.97	−41.67
*B*_30–75_ *C*_1_	−6.88	−29.64	−50.00	−0.21	53.48	−35.00
*B*_30–75_ *C*_2_	−2.39	−50.39	−104.17	3.04	44.48	−41.67
*B*_30–75_ *C*_3_	−3.67	−25.00	−82.14	1.88	46.28	−89.29
*B*_30–75_ *C*_4_	−10.55	−442.80	20.69	−8.02	−6.36	27.59
*B*_30–75_ *C*_5_	−14.80	21.26	−24.00	−10.73	69.01	−4.00
*B*_30–75_ *C*_6_	2.67	−86.94	−34.29	8.52	25.10	8.57
*B*_50–150_ *C*_1_	−4.41	−13.79	−15.00	14.40	46.01	50.00
*B*_50–150_ *C*_2_	−6.60	14.53	−17.14	29.50	53.66	62.86
*B*_50–150_ *C*_3_	−15.10	18.17	−15.56	1.50	54.17	42.22
*B*_50–150_ *C*_4_	−16.82	32.23	33.33	−12.98	68.97	6.67
*B*_50–150_ *C*_5_	−8.81	59.72	−40.00	14.46	78.78	36.00
*B*_50–150_ *C*_6_	−12.29	35.90	20.37	−0.03	74.59	40.74

**Table 9:** Comparison among our BOCA with normative knowledge and NSGA-II and PAES.

Instance	Δ_**S**_4__^**S**_3_^	Δ_*t*_4__^*t*_3_^	$\Delta_{\left|{\hat{X}}_{4}\right|}^{\left|{\hat{X}}_{3}\right|}$	Δ_**S**_5__^**S**_3_^	Δ_*t*_5__^*t*_3_^	$\Delta_{\left|{\hat{X}}_{5}\right|}^{\left|{\hat{X}}_{3}\right|}$
*A*_10–25_ *C*_1_	0.01	−704.79	0.00	0.01	−122.75	0.00
*A*_10–25_ *C*_2_	0.00	−758.52	0.00	0.00	−123.86	0.00
*A*_10–25_ *C*_3_	−0.01	−910.33	0.00	−0.01	−77.17	0.00
*A*_10–25_ *C*_4_	0.00	−1550.78	0.00	0.00	−58.03	0.00
*A*_10–25_ *C*_5_	0.02	−940.48	0.00	0.02	−125.00	0.00
*A*_10–25_ *C*_6_	−0.35	−787.10	−12.50	−0.35	−106.45	−12.50
*A*_30–75_ *C*_1_	−3.10	−105.93	−12.50	0.42	37.07	0.00
*A*_30–75_ *C*_2_	−2.74	−124.92	−86.96	−1.50	−4.01	−78.26
*A*_30–75_ *C*_3_	−2.22	−128.15	−104.76	−0.14	1.31	−38.10
*A*_30–75_ *C*_4_	−19.04	−95.21	−100.00	−16.41	45.31	−100.00
*A*_30–75_ *C*_5_	−2.03	−131.64	−25.00	0.73	2.46	−3.13
*A*_30–75_ *C*_6_	−0.30	−175.00	−157.14	20.37	4.66	14.29
*A*_50–150_ *C*_1_	−18.37	0.04	−59.26	−10.56	41.91	7.41
*A*_50–150_ *C*_2_	−18.37	40.88	−44.44	−4.85	71.14	14.81
*A*_50–150_ *C*_3_	−6.89	−39.78	−41.67	3.78	22.81	−8.33
*A*_50–150_ *C*_4_	−8.46	−79.19	0.00	−4.39	14.20	11.76
*A*_50–150_ *C*_5_	−20.03	−4.84	−90.48	−12.86	46.38	−9.52
*A*_50–150_ *C*_6_	−20.31	5.84	−46.15	1.13	49.23	−3.85

**Table 10:** Comparison among our BOCA with normative knowledge and NSGA-II and PAES.

Instance	Δ_**S**_4__^**S**_3_^	Δ_*t*_4__^*t*_3_^	$\Delta_{\left|{\hat{X}}_{4}\right|}^{\left|{\hat{X}}_{3}\right|}$	Δ_**S**_5__^**S**_3_^	Δ_*t*_5__^*t*_3_^	$\Delta_{\left|{\hat{X}}_{5}\right|}^{\left|{\hat{X}}_{3}\right|}$
*B*_10–25_ *C*_1_	0.00	−634.18	0.00	0.00	−102.55	0.00
*B*_10–25_ *C*_2_	−0.74	−1017.19	−25.00	−0.74	−216.41	−25.00
*B*_10–25_ *C*_3_	−1.81	−462.45	−8.33	−1.81	−89.08	−8.33
*B*_10–25_ *C*_4_	−0.21	−686.94	0.00	−0.21	−98.20	0.00
*B*_10–25_ *C*_5_	−11.96	−854.59	−50.00	−11.96	−96.22	−50.00
*B*_10–25_ *C*_6_	−2.79	−414.69	−13.33	−2.79	−60.82	−13.33
*B*_30–75_ *C*_1_	−9.56	−112.77	−15.38	−2.72	23.64	−3.85
*B*_30–75_ *C*_2_	−2.13	−72.90	−63.33	3.28	36.17	−13.33
*B*_30–75_ *C*_3_	−1.07	−96.93	−82.14	4.34	15.37	−89.29
*B*_30–75_ *C*_4_	−7.80	−198.95	14.81	−5.34	41.42	22.22
*B*_30–75_ *C*_5_	−11.09	−56.27	16.22	−7.15	38.50	29.73
*B*_30–75_ *C*_6_	4.87	−158.41	−95.83	10.59	−3.54	−33.33
*B*_50–150_ *C*_1_	−4.22	−204.86	−27.78	14.56	−44.63	44.44
*B*_50–150_ *C*_2_	−5.34	−62.92	−70.83	30.33	11.67	45.83
*B*_50–150_ *C*_3_	−11.42	−33.88	−300.00	4.64	25.03	−100.00
*B*_50–150_ *C*_4_	−11.61	−330.96	41.18	−7.93	−97.35	17.65
*B*_50–150_ *C*_5_	−16.76	−377.39	−29.63	8.21	−151.45	40.74
*B*_50–150_ *C*_6_	−6.20	−82.12	−26.47	5.40	27.79	5.88

## Appendix

### Result Tables

In this appendix section, obtained results are presented. Columns *S*_{·}_ show the *S* value obtained by algorithm {·} as %. Algorithms are indexed as follows. The original BOCA algorithm is indexed by {1}. BOCA algorithms using circumstantial and normative knowledge are indexed by {2} and {3}, respectively. Finally, the other EAs considered in this paper, namely, NSGA-II and PAES, are indexed by {4} and {5}, respectively. Columns *t*_{·}_ show the time obtained by each algorithm in seconds. Columns $\left|{\hat{X}}_{\left\{\cdot \right\}}\right|$ show the number of efficient solutions found by the corresponding algorithm. Finally operator Δ_{··}_^{·}^ shows a value that is equivalent to ({·}−{··})/{·} × 100.
